# Impact of the coronavirus disease 2019 (COVID-19) pandemic on the adherence to hand hygiene practice in hospitals—Data from a Swiss national surveillance system

**DOI:** 10.1017/ice.2022.308

**Published:** 2023-09

**Authors:** Susanne Rüfenacht, Philipp Kohler, Rolf Kuhn, Domenica Flury, Andreas F. Widmer, Matthias Schlegel, Swissnoso

**Affiliations:** 1 Cantonal Hospital St Gallen, Division of Infectious Diseases and Hospital Epidemiology, St Gallen, Switzerland; 2 Swissnoso, National Center for Infection Control, Bern, Switzerland

## Abstract

In >100,000 observations across Swiss acute-care hospitals, hand hygiene (HH) adherence significantly increased during the first coronavirus disease 2019 (COVID-19) wave. However, despite persisting COVID-19 activity, HH adherence returned to prepandemic levels over a 2-year observation period. These results indicate that training and support remains challenging.

Hand hygiene (HH) of healthcare workers (HCWs) is the most effective measure to prevent cross transmission of microorganisms.^1^ HH adherence in hospitals improves during influenza season, probably due to increased awareness and fear of contracting the disease among HCWs.^2,3^ However, short- and long-term effects of the coronavirus disease 2019 (COVID-19) pandemic on HH adherence are unknown. We examined trends in HH adherence during the COVID-19 pandemic by analyzing data from the nationwide registry for HH.

## Methods

### Participants and setting

**Fig. 1.**
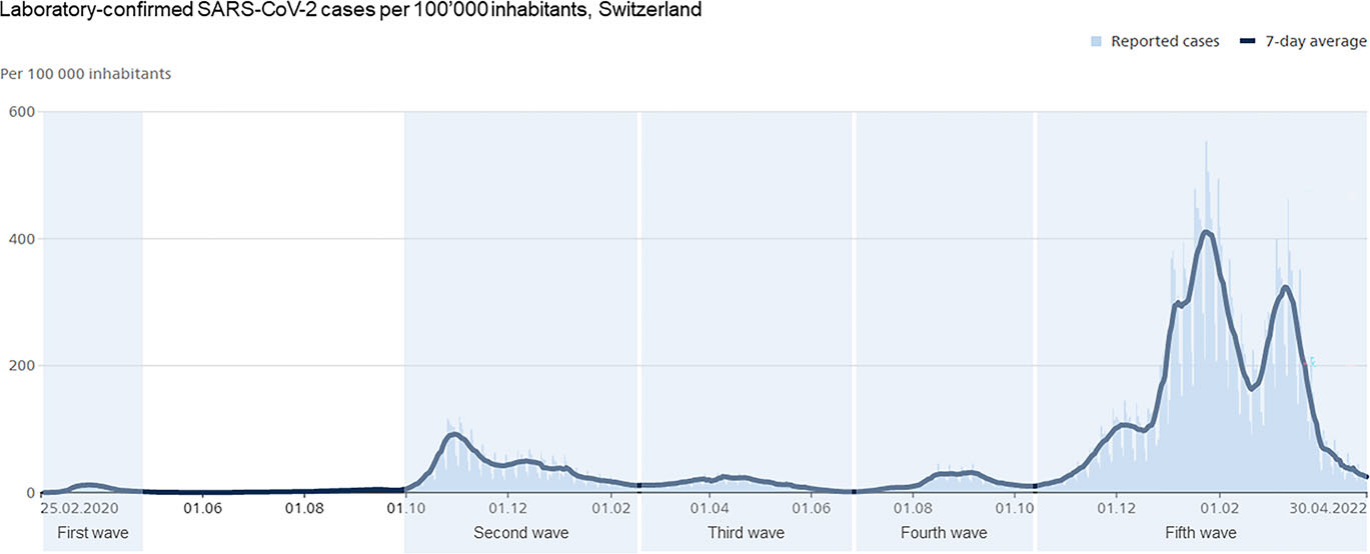
Laboratory-confirmed COVID-19 cases per 100,000 population in Switzerland. Light blue lines indicate reported cases; the bold, dark-blue curve reflects the 7-day average. First wave: February 25, 2020–April 30, 2020. Second wave: October 1, 2020–February 14, 2021. Third wave: February 15, 2021–June 20, 2021. Fourth wave: June 21, 2021–October 10, 2021. Fifth wave: October 11, 2021–April 30, 2022.

Adherence to HH practice is monitored nationwide in Switzerland using the mobile software application CleanHands.^4^ Data from individual hospitals are automatically transferred to a mainframe computer that allows the user to benchmark the result with hospitals of similar size. The program was developed by the Swiss National Centre for Infection Prevention (https://www.swissnoso.ch) within the clean care program. Only representative data from adult acute-care hospitals with at least 50 observed HH opportunities restricted to the inpatient setting were included. Correct adherence was defined and assessed according to the World Health Organization (WHO) Five Moments of HH.^5^ Indications before touching a patient and before clean or aseptic procedures were referred to as indications related to patient safety, and indications after body fluid exposure, after touching a patient, after touching patient surroundings as indications related to HCW safety. Adherence to HH practice during pandemic waves was compared to the baseline (ie, January 1, 2019, through February 24, 2020) or reference period. The COVID-19 waves were defined according to the Swiss national COVID-19 surveillance program^6^ and lasted from February 25, 2020, until April 30, 2020 (first wave); from October 1, 2020, to February 14, 2021 (second wave); from February 15, 2021, to June 20, 2021 (third wave); from June 21, 2021, to October 10, 2021 (fourth wave); and from October 11, 2021, to April 30, 2022 (fifth wave) (Fig. 1).

### Statistical analysis

We calculated HH adherence as the percentage of correct observations among all observations per pandemic wave; 95% confidence intervals were calculated using the Wilson score method. *P* values for the comparison of adherence between time points were derived from 2-sample tests for binomial proportions. Subgroup analyses by HH indication, profession, and ward type [ie, intensive care units (ICUs) vs non–intensive care units (non-ICUs)] were also performed. As additional analysis, adherence was also analyzed on the institutional level, accounting for potential differences among participating institutions. Analyses were performed using SPSS version 22 software (IBM, Armonk, NY).

## Results

**Fig. 2.**
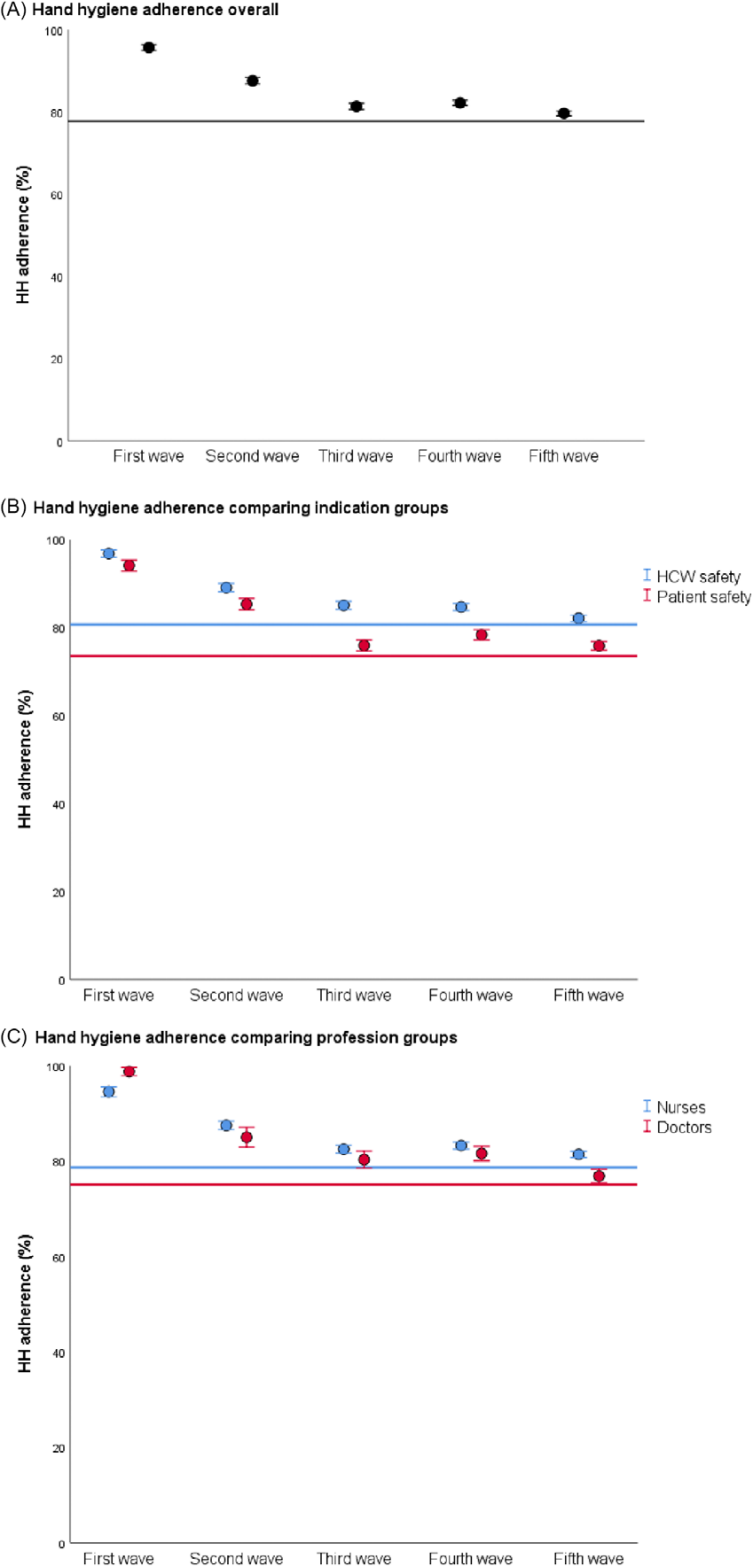
Hand hygiene adherence (%, confidence interval) of healthcare workers during COVID-19 waves in Switzerland. (A) Horizontal black line, (B) blue line, and (C) red line show corresponding baseline HH adherence during reference period (January 1, 2019–February 24, 2020).

From January 2019 until April 2022, 104,778 HH opportunities were observed in 71 hospitals (median, 665 observations; IQR, 267–1,936). Of all observations, 39% took place during the reference period and 61% during the pandemic. Overall adherence abruptly increased with the beginning of COVID-19 activity in Switzerland from 77.7% up to 95.7% (*P* < .001) and was highest during the first wave, followed by a gradual decrease toward the baseline (Fig. 2A). A similar pattern was observed on the institutional level, although we detected considerable variation among institutions (Supplementary Fig. 1 online). Adherence for indications related to both “patient safety” and “HCW safety” increased during the first COVID-19 wave from 73.5% to 94.1% (*P* < .001) and from 80.7% to 96.8% (*P* < .001), respectively. The subsequent decline was more pronounced in HH adherence related to patient safety than to HCW safety (Fig. 2B). Regarding professions, the increase in HH adherence during the first wave was more pronounced for physicians compared to nurses: from 75.1% to 98.8% (*P* < .001) versus from 78.6% to 94.5% (*P* < .001) (Fig. 2C). HH adherence was similar on ICU and non-ICU wards.

## Discussion

HH increased during the first phase of the COVID-19 pandemic in both the patient safety and HCW safety indications. The effect was more pronounced for physicians than for nurses. The pandemic may represent a powerful trigger to ameliorate HH adherence even under staff shortages and a stressful hospital environment. Trends in HH improvement with subsequent decline were observed in different parts of the world during the first COVID-19 pandemic wave.^7–9^ However, these studies used electronic surveillance methods, and our study results were directly observed at HH opportunities. The latter is still considered the gold standard but may overestimate HH adherence by observing HCWs on site. Lack of increase in HH adherence during the pandemic was also reported.^10^ Our study showed a decline in HH adherence toward the baseline over the 2-year observation period despite ongoing COVID-19 activity. Various reasons can potentially explain the decline: Overestimation of the transmission by contact at the beginning of the pandemic, introduction of vaccination, habituation to the situation, and/or changes in workload during the following waves.

The analysis of directly observed nationwide data regarding HH adherence over >2 years during the COVID-19 pandemic is a particular strength of the study. As a study limitation, participating institutions determined when and on which departments observations were made, introducing potential selection bias; furthermore, it is unknown whether observations were made on COVID-19 or non–COVID-19 wards. A better understanding of the mechanisms behind the observed behavioral changes could contribute to sustainable improvement of HH adherence outside the pandemic setting.

## Supplementary material

For supplementary material accompanying this paper visit https://doi.org/10.1017/ice.2022.308. click here to view supplementary material
