# Supplementary material for: Impact of the coronavirus disease 2019 (COVID-19) pandemic on the adherence to hand hygiene practice in hospitals—Data from a Swiss national surveillance system
Source: Infect Control Hosp Epidemiol. 2023 Jan 10;44(9):1522–5. doi: 10.1017/ice.2022.308 (PMC10507510; doi:10.1017/ice.2022.308)
Supplement: Supplementary file 1 [file S0899823X22003087sup.zip › S0899823X22003087sup002.docx]

Supplementary Fig. 1. Hand hygiene adherence (% and confidence interval) of participating institutions. **F**irst wave: February 25, 2020–April 30, 2020. Second wave: October 1, 2020–February 14, 2021. Third wave: February 15, 2021–June 20, 2021. Fourth wave: June 21, 2021–October 10, 2021. Fifth wave: October 11, 2021–April 30, 2022.
