# Supplementary figures and images for: Impact of the coronavirus disease 2019 (COVID-19) pandemic on the adherence to hand hygiene practice in hospitals—Data from a Swiss national surveillance system
Source: Infect Control Hosp Epidemiol. 2023 Jan 10;44(9):1522–5. doi: 10.1017/ice.2022.308 (PMC10507510; doi:10.1017/ice.2022.308)

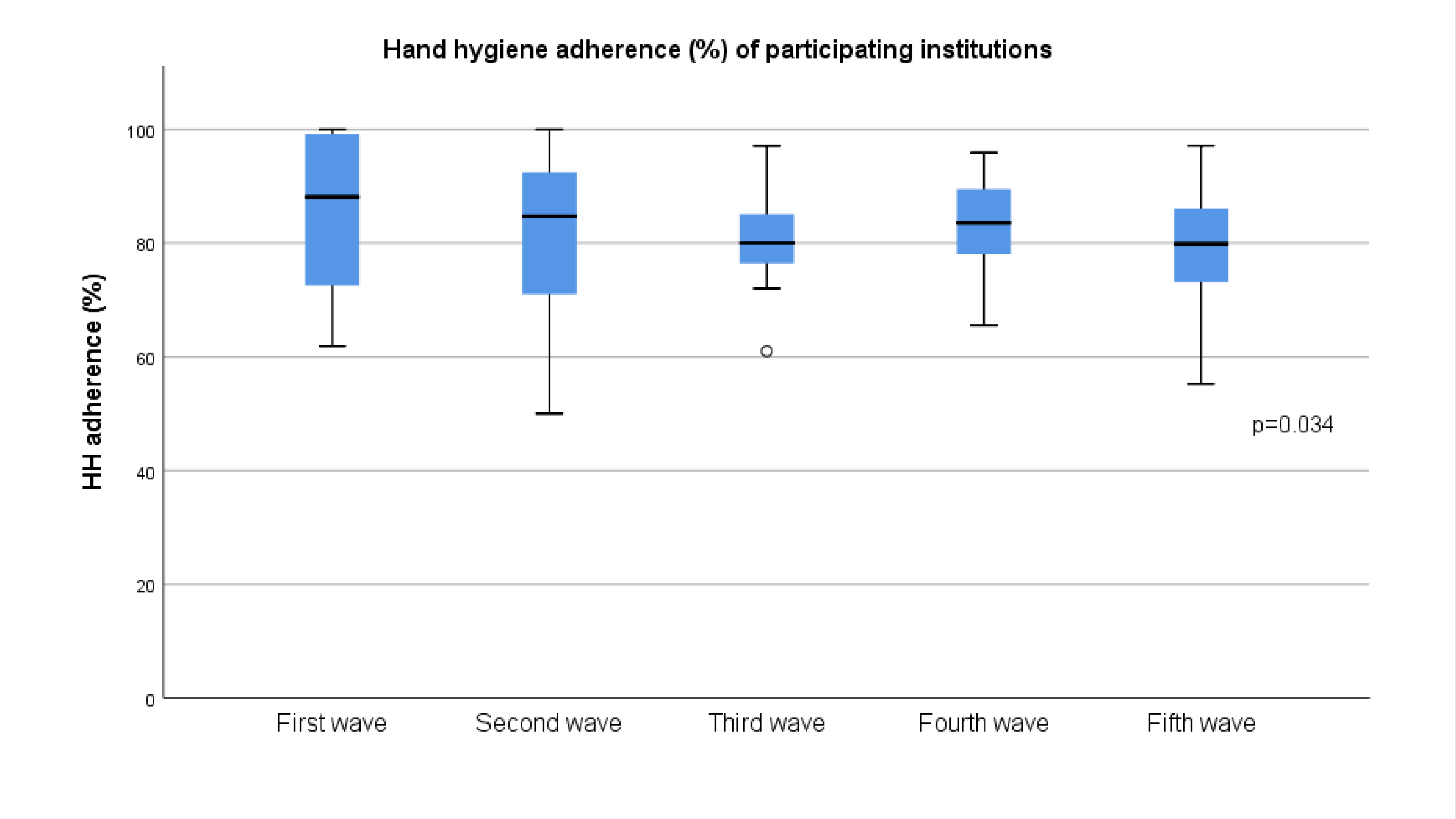

Supplement: Supplementary file 1 [file S0899823X22003087sup.zip › S0899823X22003087sup001.tif]
